# Quality Improvement Project on Uned Gobaith, the Mother and Baby Unit: Improving Monitoring and Records of Response to Psychotropic Medication on the MBU

**DOI:** 10.1192/bjo.2026.11462

**Published:** 2026-06-30

**Authors:** Katherine Ferrer, Shambhavi Pranoy, Nermeen Ahmed, Angharad Piette

**Affiliations:** Swansea Bay University Health Board, Swansea, United Kingdom

## Abstract

**Aims:**

To audit, and then work with the MDT to identify ways to improve compliance with Perinatal Quality Network (PQN) standards. The standards were regarding pharmacological management, including the requirements when starting new medication, and weekly reviews for regular medication and PRN usage. The agreed measure is the percentage of time that these medications are monitored and this is recorded appropriately. As per the PQN, the acceptable level would be 90% plus in order to meet Sustainable Service Accreditation.

**Methods:**

A month of records were examined for six patients including MDT minutes,reviews and the prescribing interface. Data was collected to review whether monitoring, therapeutic response and discussions about medication met the PQN standards. After this cycle, the MDT met to implement changes to the weekly review and ward documentation to improve compliance. A further month of records following this change were examined.

**Results:**

Following changes, monitoring compliance increased. Therapeutic response monitoring for regular medication improved from 69.1% in cycle 1 to 93.6% in cycle 2, safety assessment from 44.9% to 92%, side-effect discussions rising from 22.4% to 98% and adherence recording going from 78.5% to 96%. No new medication reviews were conducted during cycle 1, so there was no baseline data, however treatment goal, risks and benefits were discussed in all reviews in cycle 2. Timescale however was not specified in any of the three reviews. Changes to PRN monitoring were also seen with weekly reviews conducted 36.7% of the time in cycle 1, increasing to 67.5% in cycle 2.

**Conclusion:**

Discussion and involvement of the MDT to understand need for monitoring and aim of accreditation led to positive changes in pharmacological management, contributing to sustainable principles. The use of the PQN Quality standards to help guide MDT discussion and patient reviews has been successful in ensuring best-quality care is provided.

